# *Magnolia officinalis* enhanced immune responses and the resistance to *Vibrio harveyi* infection in pearl gentian groupers

**DOI:** 10.3389/fvets.2025.1603997

**Published:** 2025-06-18

**Authors:** Yilin Zhang, Yuhao Li, Xinlan Xu, Luxi Xu, Weifu Li, Zhen Gan, Yishan Lu

**Affiliations:** ^1^Guangdong Provincial Key Laboratory of Aquatic Animal Disease Control and Healthy Culture and Key Laboratory of Control for Disease of Aquatic Animals of Guangdong Higher Education Institute, College of Fishery, Guangdong Ocean University, Zhanjiang, China; ^2^Guangdong Provincial Engineering Research Center for Aquatic Animal Health Assessment and Shenzhen Public Service Platform for Evaluation of Marine Economic Animal Seedings, Shenzhen Institute of Guangdong Ocean University, Shenzhen, China

**Keywords:** *Magnolia officinalis*, *Vibrio harveyi* ZJ0603, pearl gentian groupers, immune response, immunostimulant

## Abstract

*Magnolia officinalis* (MO) is a kind of traditional Chinese herbs, which has been studied for thousands of years in Chinese pharmacopoeia. In recent years, MO has been reported as an antibacterial agent in aquaculture, but the antibacterial properties of MO against *Vibrio harveyi* infection in fish remains unexplored. In this study, the effects of MO treatment on immune responses and the resistance to *V. harveyi* infection were detected in pearl gentian groupers. The results revealed that the expression levels of immune-related genes (*IL-12, TLR2, TLR5S, CD4, MHC-Iα*, and *IFN-γ*) in spleen, head kidney, liver and thymus, and the enzyme activities of CAT, SOD, LZM, and total serum protein in serum were significantly up-regulated at most of time points in MO -treated groupers. After being challenged with *V. harveyi* ZJ0603 at 28 days post-injection, the survival rate (SR) of groupers were 50.0, 60.0, 73.3, and 66.7% in MO groups at different concentrations, respectively, indicating that MO administration could improve the resistance to *V. harveyi* infection in groupers. The present study revealed that MO can be considered as a promising immunostimulant to induce the immune responses against *V. harveyi* infection in marine fishes.

## 1 Introduction

Infectious diseases caused by *Vibrio* spp. pose a serious threat to the health of humans and animals, and several species of *Vibrio* are known to cause illnesses in marine animals, including marine fishes, crabs, and shrimps (1). *Vibrio harveyi* is considered as a severe pathogen affecting a large number species of marine fishes, and it is known to induce gastroenteritis, necrotizing enteritis, nodules on the operculum, scale drop and muscle necrosis, skin ulcers, and tail rot in different species of fish (2). Predominantly cultured in tropical and subtropical regions, the pearl gentian grouper (*E. fuscoguttatus* × *E. lanceolatus*) is an important economic species of marine fishes. Nonetheless, grouper aquaculture has been devastated by multiple bacterial diseases, one of which is caused by *V. harveyi* infection (3). Several studies shown that infection of *Vibrio* diseases caused by *V. harveyi*, *Vibrio vulnificus*, and *Vibrio alginolyticus*, et al., resulted in serious economic losses in aquaculture industry of pearl gentian grouper (4). Therefore, it is very necessary to find a way to resist *Vibrio* disease of groupers. Antibiotics are widely used to combat these diseases, but their use has led to the rise of antibiotic resistance and food safety concerns (5, 6).

Chinese herbal medicine is a natural drug derived from plants, animals or minerals in nature, processed by traditional Chinese medicine theory and used for the prevention and treatment of diseases. In grouper fish farming industry, several Chinese herbal medicine or extract were used for disease prevention and improving the survival rates, such as *Astragalus membranaceus*, *Spatholobus suberectus*, *Phellodendron amurense*, *Eclipta prostrata*, *Ganoderma lucidum* polysaccharides, et al. (4, 7–9).

Known as “Hou po” in Chinese, *Magnolia officinalis* is a kind of Chinese herbal medicine that has been widely used in Asia for about 2000 years (10). Functionally, *M. officinalis* exerts a wide range of pharmacological effects on different organs/tissues in mammals (10). A large number of components have been found in *M. officinalis*, and honokiol (3,3′-diallyl-2,2′-dihydroxybiphenyl) and magnolol (5,5′-diallyl-2,2′-dihydroxybiphenyl) are identified as two important active components known for their cooperative antioxidant properties (11). Importantly, it is revealed that *M. officinalis* or its active components (magnolol and honokiol) not only have powerful directly antiviral (12–14), antibacterial (15–18), antiparasitic (19, 20), and antifungal (21) activities, also effectively improve immunity in different fish species, such as *Ctenopharyngodon idella* (12), *Carassius auratus* (15), *Micropterus Salmoides* (16), et al. However, the antibacterial properties of MO against *V. harveyi* infection remains unexplored in fish (22).

In this study, we aim to explore whether MO can be used as an effective immunostimulant against *Vibrio* spp. infection in fish. In the present study, the protective efficacy of MO against *V. harveyi* infection was evaluated in pearl gentian groupers. The immune-related genes including *IL-12, TLR2, TLR5S, CD4, MHC-Iα*, and *IFN-γ*, were tested in immune organs (spleen, head kidney, liver and thymus), and the enzyme activities of CAT, SOD, LZM, and total serum protein were also detected in serum. The findings indicated that MO represent a promising immunostimulant to induce the immune responses against *V. harveyi* infection in groupers, and a therapeutic agent to control Vibriosis in aquaculture.

## 2 Materials and methods

### 2.1 Bacterial strains and fish

*Vibrio harveyi* ZJ0603 was isolated from diseased grouper in Guangdong, China and stored in our laboratory, and was cultured in tryptic soy broth (TSB) in 28°C for the following experiment. The pearl gentian groupers, weighing 50.0 ± 5.0 g, were purchased from Donghai Island Fish Farm in Zhanjiang. These fish were kept in an oxygenated seawater tank and were fed twice daily. The animal study was reviewed and approved by Guangdong Provincial Key Laboratory of Pathogenic Biology and Epidemiology for Aquatic Economic Animals Ethics Committee (GDOU2023006).

### 2.2 MO preparation and fish treatment

*Magnolia officinalis* extract was purchased from Shanxi Yijunjian Biotech Co., LTD. MO extract was dissolved in 0.85% normal saline, and the solution was then filtered through the millipore membrane (0.45 mm) at adjusted concentrations of 2, 4, 6, and 8 mg/mL. A total of 450 fish were randomly divided into PBS, MO2, MO4, MO6, and MO8 groups, with 90 fish in each group. Fish were intraperitoneally injected with 100 μL of 2, 4, 6, and 8 mg/mL MO in MO2, MO4, MO6, and MO8 groups, respectively, while individuals was with 100 μL PBS in PBS group (control group). Spleen, head kidney, liver and thymus were collected at 2 and 4 w, and blood was collected from 1 to 6 weeks post-treatment (3 fish per group in per time point). The blood was stored overnight at 4°C and was centrifuged at 3500 rpm for 30 min to collect serum. Serum, spleen, head kidney, liver and thymus were stored at −80°C until further use.

### 2.3 Survival rate (SR) of MO-treated grouper after *V. harveyi* infection

Four weeks after injection, fish from PBS, MO2, MO4, MO6 and MO8 groups (90 fish per group) were intraperitoneally injected with 100 μL of *V. harveyi* ZJ0603 at a concentration of 5.01 × 10^6^ CFU/mL (as used in this study). Subsequently, the fish was monitored for 14 days to calculate the SR using Kaplan–Meier method (23). To ensure that the fish were killed by *V. harveyi*, organs/tissues from randomly selected dead individuals were streaked on TCBS plates to identify the bacterial species. After 16S rDNA testing (Table 1), the PCR product was sent to Sangon Biotech to identify *V. harveyi* (Supplementary Figure 1).

**Table 1:** Primers listed in this study.

Primer name	Primer sequence (5′ − 3′)
Bacteria identification	
16 s rDNA-F	AGAGTTTGATCMTGGCTCAG
16 s rDNA-R	GGTTACCTTGTTACGACTT
qRT-PCR	
IL-12-F	GTGGATGCCAGCGGTCAA
IL-12-R	GGAAATGCTCCGTCGTCA
TLR2-F	CCCACAATGGATTCACCAG
TLR2-R	AAAGATCAAGACTCAAGGCACTG
TLR5S-F	TGTTTCCCAAAACAATGTGA
TLR5S-R	CATGACCCAGAACACCAATG
CD4-F	TTGCGGTGCAAAATCCACTG
CD4-R	TGCCATCAGTCCAGGACAAC
MHC-1α-F	GCCGCCACGCTACAGGTTTCTA
MHC-1α-R	TCCATCGTGGTTGGGGATGATC
IFN-γ2-F	CAGCAATGGTGAGGTGGCA
IFN-γ2-R	TTTGCTCTGGATGATAGGGTC
β-actin-F	AACAACCACACACCACACATTTC
β-actin-R	TGTCTCCTTCATCGTTCCAGTTT

### 2.4 Quantitative real-time PCR (qRT-PCR) analysis

Total RNA was extracted from organs/tissues by using the TransZol Up Plus RNA Kit, and was used for the cDNA synthesis by using Takara’s EasyScript® One-Step gDNA Removal and cDNA Synthesis SuperMix from China. qRT-PCR analysis was then conducted to detect the expression of well-studied immune-related genes, including *IL-12, TLR2, TLR5S, CD4, MHC-Iα*, and *IFN-γ* (24–29), with *β*-actin as the reference gene. The reaction condition of qRT-PCR was as follows: preincubation at 94°C for 300 s, 3-step amplification including 40 cycles of 94°C for 10s, 60°C for 15 s, and 72°C for 20s, with melting and cooling performed finally. The data were analyzed with the 2^−ΔΔCt^ method as described in the previous studies (30, 31).

### 2.5 Enzyme activity and total serum protein (TP) in serum

The experimental measurements of catalase (CAT), lysozyme (LZM), superoxide dismutase (SOD), and total serum protein (TP) levels in serum were conducted by using a visible light-based hydrogen peroxide assay kit for catalase, a lysozyme assay kit, a superoxide dismutase assay kit, and a total protein quantification assay kit, respectively. All kits were sourced from the Nanjing Jiancheng Bioengineering Institute in China.

### 2.6 Statistical analysis

Data were statistics by one-way analysis of variance (ANOVA), followed by using SPSS 26.0 software with Duncan’s new multiple range test. Data were shown as mean ± SE, and significant differences (*p* < 0.05) are indicated by different letters (a–d). “n = 3” means three biological replicates.

## 3 Results

### 3.1 Protection of pearl gentian groupers from *Vibrio harveyi* infection by MO treatment

**Figure 1:**
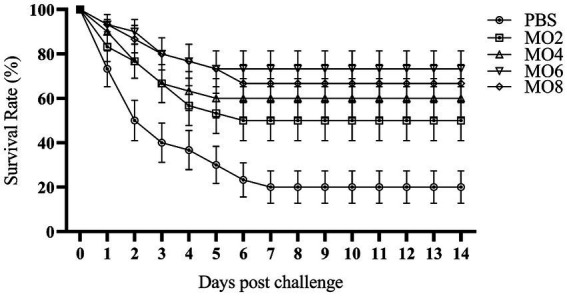
Survival rate of pearl gentian groupers challenged with *V. harveyi* for 14 days. Significant differences (*p* < 0.05) were obviously between the groups. Error bar represent mean ± SE.

To detect the protective effect of MO in groupers against *V. harveyi* infection, the fish were monitored for 14 days to calculate the SR for all the groups. As shown in Figure 1, the SR of MO2, MO4, MO6, and MO8 groups were 50.0, 60.0, 73.3, and 66.7%, respectively, all of which were significantly higher than 26.7% in the PBS group (*p* < 0.05). These results showed that the treatment of MO facilitated the survival of fish after *V. harveyi* infection.

### 3.2 Effect of MO treatment on the expression of immune-related genes in pearl gentian groupers

**Figure 2:**
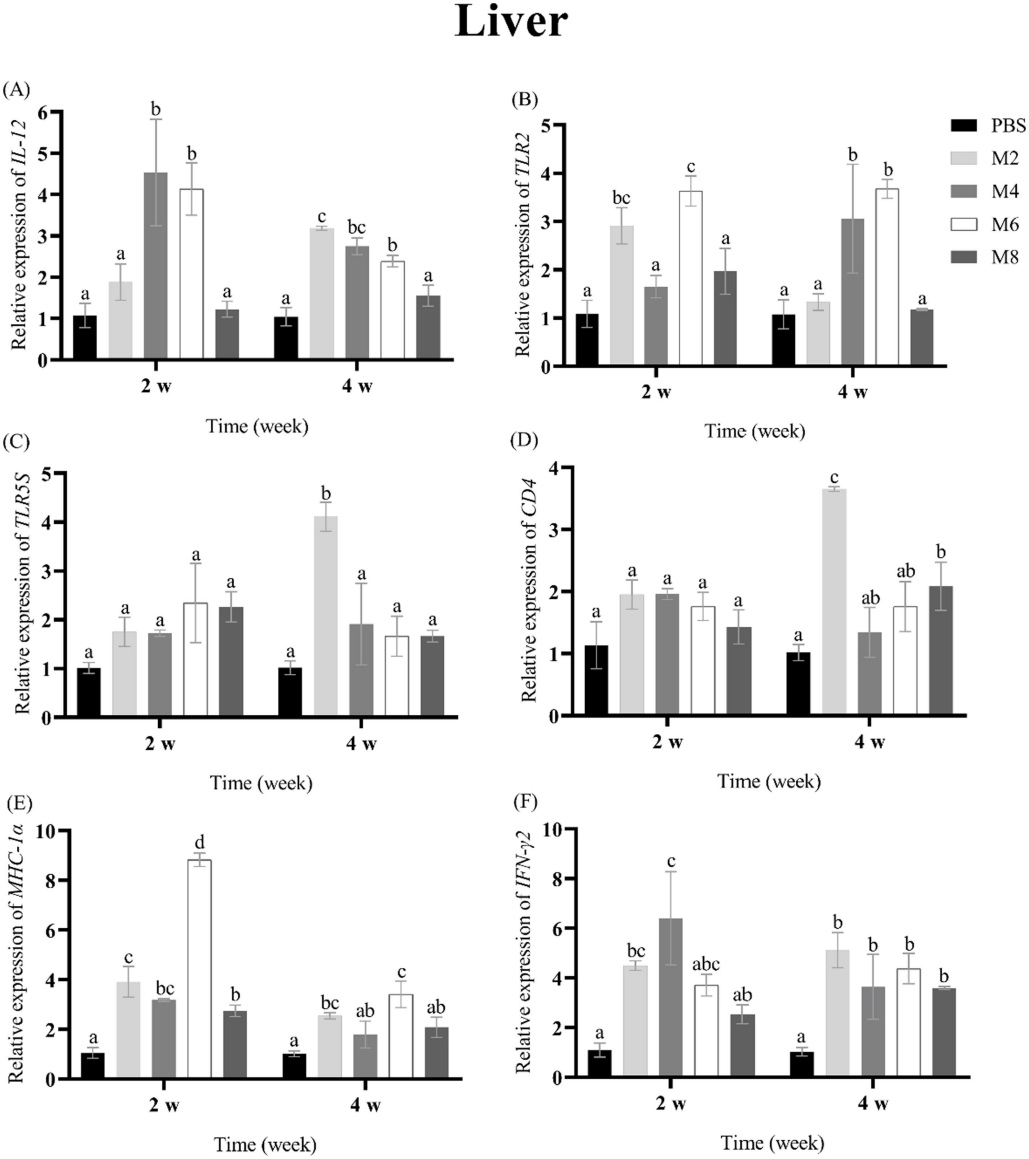
The expression levels of immune-related genes in the liver of pearl gentian groupers by qRT-PCR post-immunization. The mRNA level of each immune-related gene was normalized to that of *β*-actin and relative expression was calculated as the values of the vaccinated tissues by those of the controls. **(A)** *IL-12*, **(B)** *TLR2*, **(C)** *TLR5*, **(D)** *CD4*, **(E)** *MHC-Iα*, **(F)** *IFN-γ*. Bars represented the mean relative expression (*n* = 3). Significant differences (*p* < 0.05) are marked by different letters (a–d).

**Figure 3:**
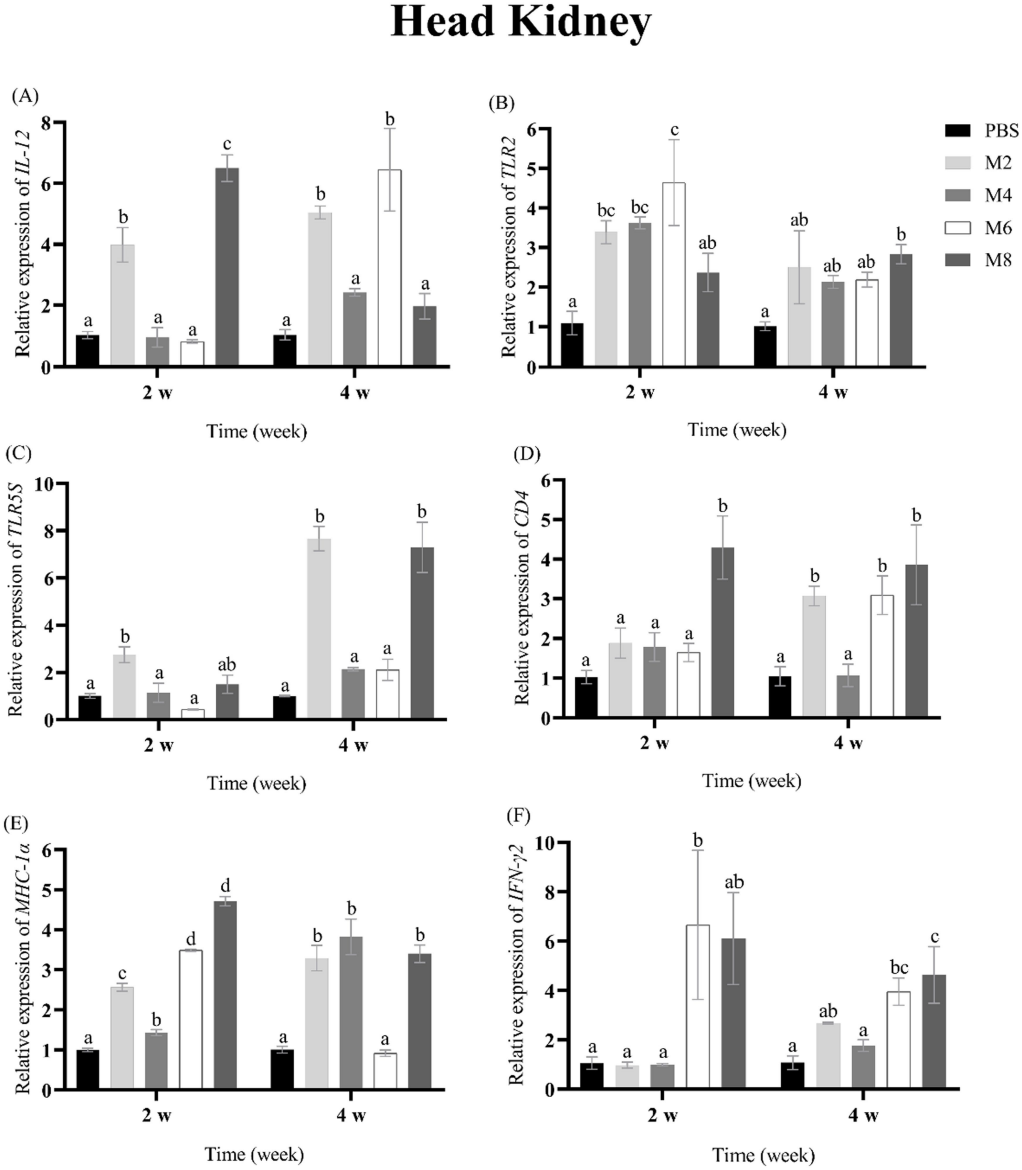
The expression levels of immune-related genes in the head kidney of pearl gentian groupers by qRT-PCR post-immunization. The mRNA level of each immune-related gene was normalized to that of β-actin and relative expression was calculated as the values of the vaccinated tissues by those of the controls. **(A)** *IL-12*, **(B)** *TLR2*, **(C)** *TLR5*, **(D)** *CD4*, **(E)** *MHC-Iα*, **(F)** *IFN-γ*. Bars represented the mean relative expression (*n* = 3). Significant differences (*p* < 0.05) are marked by different letters (a–d).

**Figure 4:**
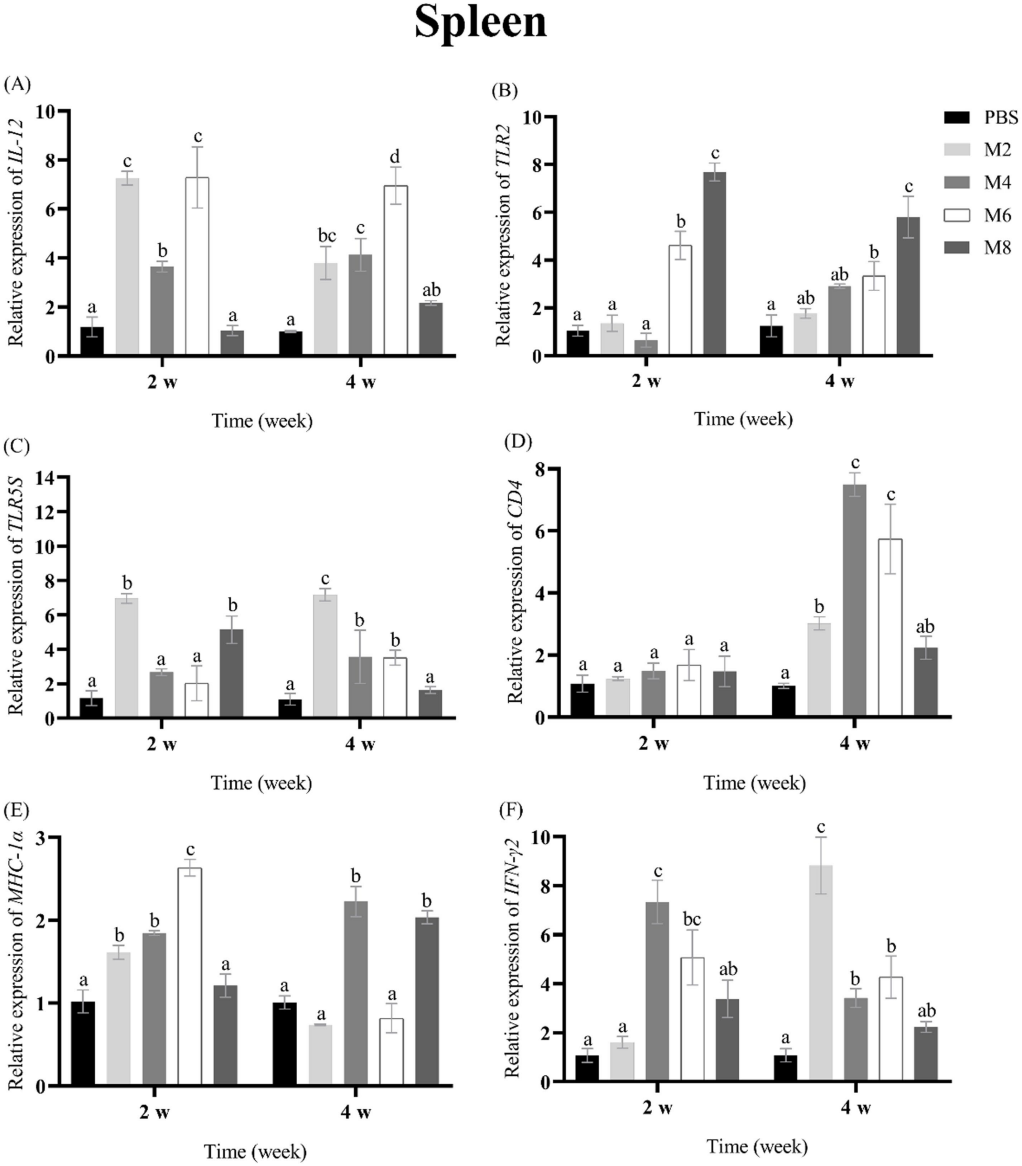
The expression levels of immune-related genes in the spleen of pearl gentian groupers by qRT-PCR post-immunization. The mRNA level of each immune-related gene was normalized to that of β-actin and relative expression was calculated as the values of the vaccinated tissues by those of the controls. **(A)** *IL-12*, **(B)** *TLR2*, **(C)** *TLR5*, **(D)** *CD4*, **(E)** *MHC-Iα*, **(F)** *IFN-γ*. Bars represented the mean relative expression (*n* = 3). Significant differences (*p* < 0.05) are marked by different letters (a–d).

**Figure 5:**
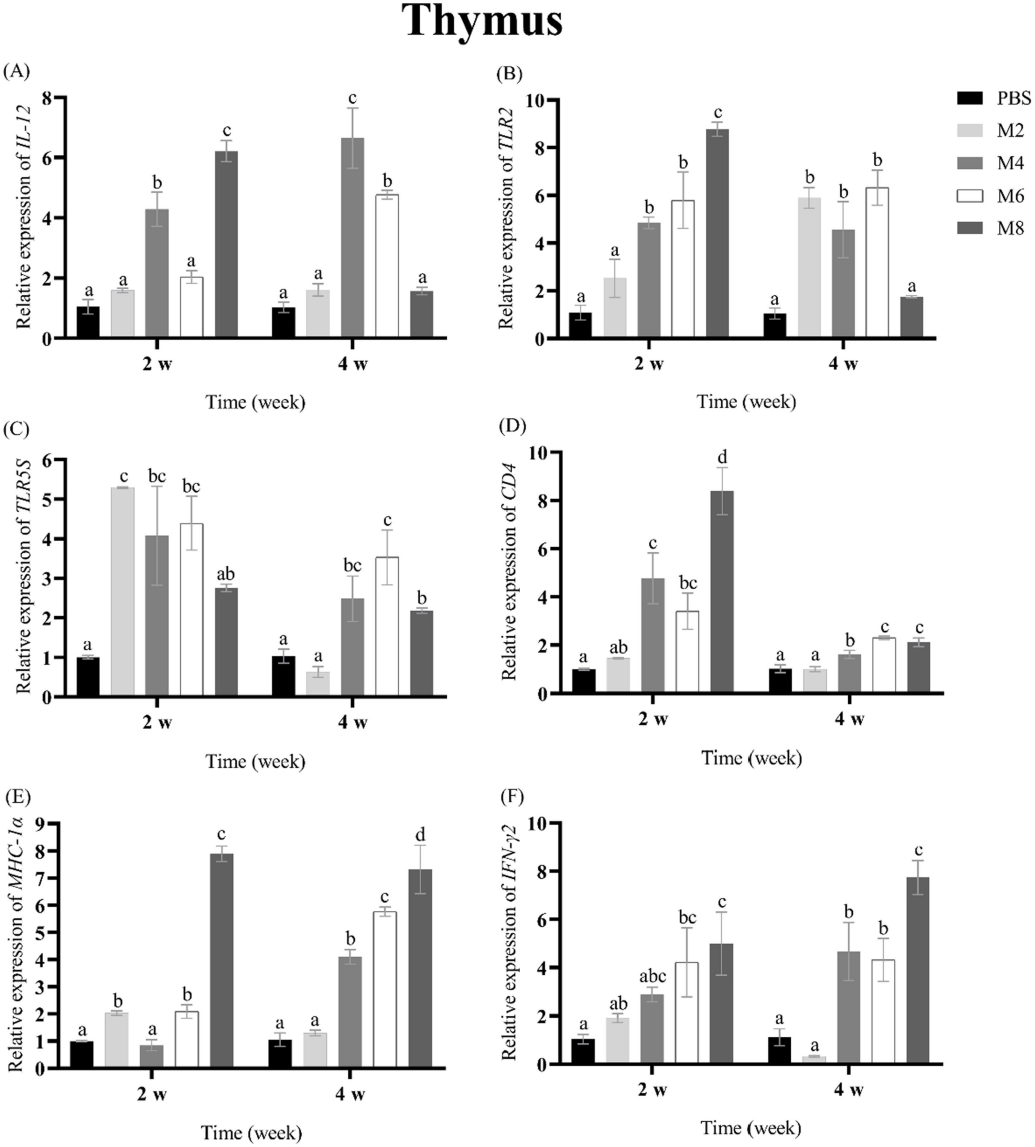
The expression levels of immune-related genes in the thymus of pearl gentian groupers by qRT-PCR post-immunization. The mRNA level of each immune-related gene was normalized to that of β-actin and relative expression was calculated as the values of the vaccinated tissues by those of the controls. **(A)** *IL-12*, **(B)** *TLR2*, **(C)** *TLR5*, **(D)** *CD4*, **(E)** *MHC-Iα*, **(F)** *IFN-γ*. Bars represented the mean relative expression (*n* = 3). Significant differences (*p* < 0.05) are marked by different letters (a–d).

To assess the expression of immune-related genes in groupers after the treatment of different concentrations of MO, the transcriptional levels of *IL-12, TLR2, TLR5S, CD4, MHC-Iα* and *IFN-γ* were measured in spleen, head kidney, liver and thymus of groupers at 2 and 4 w post-treatment (Figures 2–5). The expression pattern of *IL-12* was different in spleen, head kidney, liver, and thymus, and its expression was significantly up-regulated in spleen of MO2, MO4 and MO6 groups at 2 and 4 weeks, in head kidney of MO2 group for 2 and 4 weeks, in liver of MO4 and MO6 groups for both 2 weeks, and in thymus of MO4 group for both 2 weeks (Figures 2A, 3A, 4A, 5A). For *TLR2*, its expression was significantly up-regulated in head kidney and thymus of four groups for both 2 weeks, except MO8 group at the time-point of 4 weeks in thymus (Figures 3B, 5B). By contrast, the significant expression of *TLR2* induced by MO both 2 weeks was only observed in liver of MO6 group, and spleen of MO6 and MO8 groups (Figures 2B, 4B). For *TLR5S*, its expression was significantly up-regulated in thymus of four groups, except MO2 group at the time-point of 4 w (Figure 5C). In spleen, liver and head kidney, the expression pattern of *TLR5S* was different, with no significant difference of most of groups (Figures 2C, 3C, 4C). For *CD4*, its expression in four groups was significantly up-regulated in spleen and liver, and head kidney at 4 w time-point, except MO4 group in head kidney (Figures 2D, 3D, 4D). In thymus, the expression pattern of *CD4* was up-regulated of MO4, MO6, and MO8 groups for both 2 weeks (Figure 5D). For *MHC-Iα*, its expression was markedly induced in liver of four groups, and was differently regulated in other three organs/tissues (Figures 2E, 3E, 4E, 5E). Lastly, the expression of *IFN-γ* was significantly induced in four organs/tissues of MO6 and MO8 groups (Figures 2F, 3F, 4F, 5F).

### 3.3 Effect of MO treatment on CAT, LZM, SOD enzyme activity and total protein in serum of pearl gentian groupers

**Figure 6:**
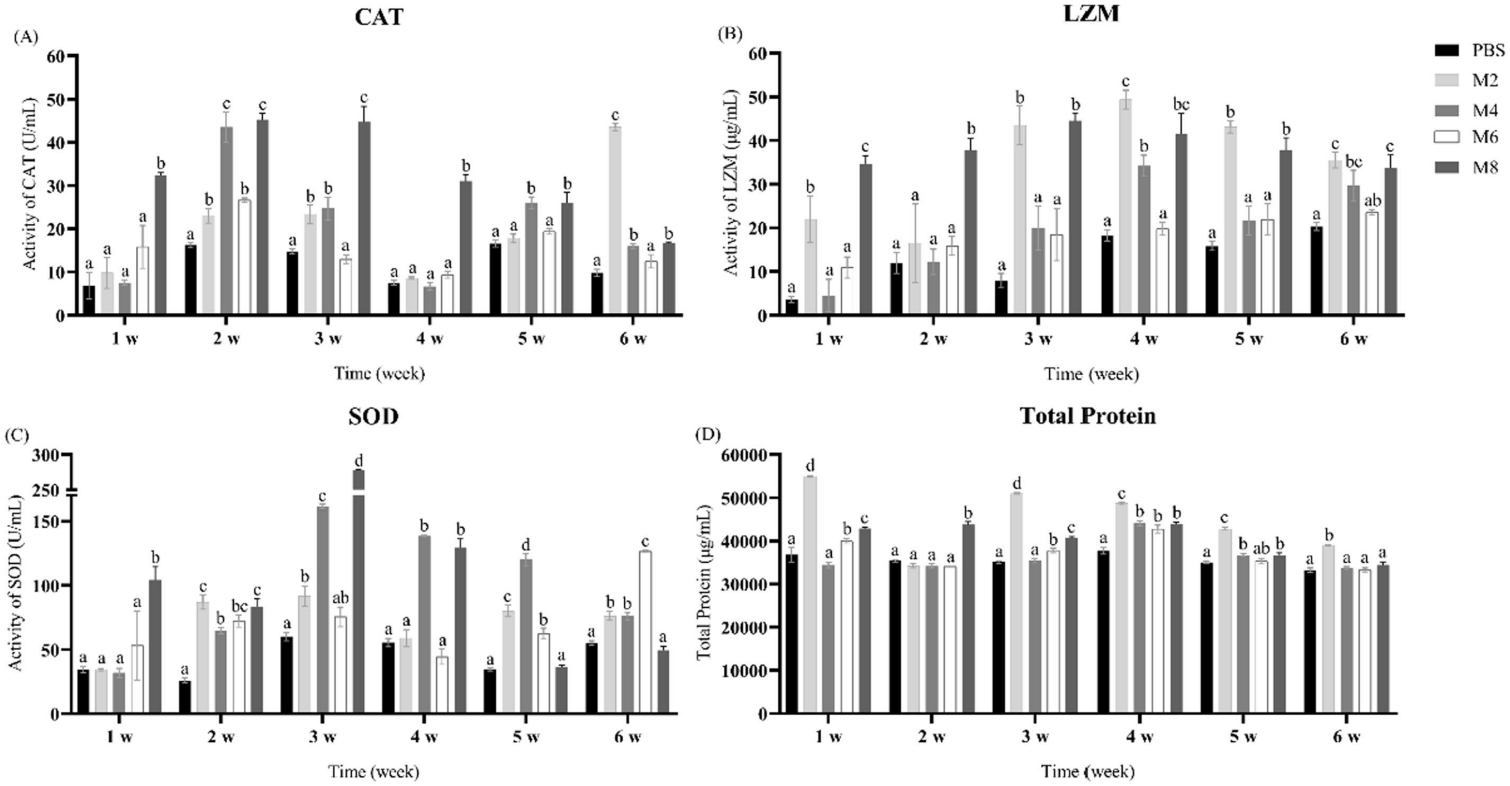
Catalase (CAT), lysozyme (LZM) and superoxide dismutase (SOD) activities and total serum protein of pearl gentian grouper from 1 to 6 w post-injection (mean ± SE; *n* = 3). **(A)** CAT activity, **(B)** LZM activity, **(C)** SOD activity, and **(D)** total serum protein. Significant differences (*p* < 0.05) are marked by different letters (a–d).

In MO8 group, the activities of CAT and LZM significantly increased at all time-points, while SOD activity and TP significantly increased only during the first 5 weeks (Figures 6A–D). By contrast, in all experimental groups, induced-expression of CAT, LZM, and SOD enzyme activity was observed for a long period of 6 weeks, and induced-expression of TP could only maintain until the fifth week.

## 4 Discussion

Immunostimulants used to prevent disease in aquaculture has attracted much attention in recent years. Chinese herbal medicine or their active ingredients as one kind of immunostimulant play roles in enhancing disease resistance of fish by regulating the non-specific and specific immune response, which have been confirmed by multiple studies. *Scutellaria baicalensis*, *A. membranaceus*, *Lycium barbarum* and *A. membranaceus* polysaccharide were confirmed to enhance the SR after infected by gram-negative bacteria and innate immunity of fish (8, 32–34). Likewise, *M. officinalis* as one of traditional Chinese herbs, and its effective ingredients magnolol and honokiol have been shown to be effective in preventing *Carassius auratus* from *Aeromonas hydrophila*, *A. veronii* and *Ichthyophthirius multififiliis* infections (15, 19), channel catfish from *A. hydrophila* infection (35), and *Micropterus Salmoides* from *Nocardia seriolae* infection (16), by improving innate immunity index and SR. The molecular mechanism of MO is mainly manifested in anti-inflammatory and anti-bacterial effects. Firstly, magnolol and honokiol inhibit excessive inflammatory responses by targeting the TLR signaling pathway or directly binding to NF-κB, thereby suppressing the expression of inflammatory factors (36, 37). Secondly, the anti-bacterial activity achieved through multiple mechanisms, including destroying the cell wall, interfering with the function of the cell membrane, and inhibiting cell growth and reproduction, et al. (36, 37). In our research, we aimed to explore whether MO could significantly enhance the immunity and against bacterium infection of fish, which providing a new idea for the prevention and control of aquatic disease.

The recent surge in Vibriosis has hindered the development of the aquaculture industry, and it was critically important to develop a sustainable and healthy approach to combat Vibriosis (38, 39). It is reported the high efficacy of traditional Chinese herbs and their active ingredients in combating Vibriosis (18, 40). *M. officinalis* is one of the traditional Chinese herbs, and its effective ingredients magnolol and honokiol have been proven to improve immunity of the fish (15, 16, 19, 35) and have direct anti-bacterial effect (16, 36). However, the antibacterial properties of MO against *V. harveyi* infection remains unexplored in fish. In this study, our data revealed that the SR of groupers treated with MO at concentrations of 2, 4, 6, and 8 mg/mL was notably increased by 50.0, 60.0, 73.3, and 66.7%, respectively, compared to 26.7% in the control group, indicating that MO administration could enhanced the resistance of groupers to *V. harveyi* infection.

Fish immune responses are a multifaceted process that hinges on the collaboration of various components, including immune organs, cells, and molecules (41, 42). For immune organs, liver is an active site of lymphopoiesis primarily involved in innate immunity, inflammation, and homeostasis (41) and spleen is considered as an important peripheral lymphoid organ in fish (41, 43, 44). Head kidney is a crucial hematopoietic organ with regulatory functions and the center of immune-endocrine interactions (41, 45, 46), and thymus is responsible for the production of T cells and the stimulation of phagocytosis in teleost fish (41, 47, 48). To detect the immune response of groupers after MO injection, the expression of *IL-12, TLR2, TLR5S, CD4, MHC-Iα*, and *IFN-γ* in four tested organs. As a potent immunoregulatory cytokine, IL-12 can activate NK cells and promote macrophages to clear phagocytic pathogens, and enhance the resistance to bacteria, viruses, and parasites (25, 28). IFN-γ is a soluble cytokine produced by T cells, macrophages, mucosal epithelial cells, and natural killer cells, and it activates the JAK–STAT pathway, leading to the induction of genes involved in both innate and adaptive immune responses (49). MHC-Iα and MHC-IIα are primarily expressed on antigen-presenting cells and bind to CD8 and CD4 on cytotoxic T lymphocyte and helper T cells, respectively, to help distinguish between self and non-self antigens and initiate an appropriate immune response (27, 29, 50). In this study, the expression of *IL-12, CD4, MHC-Iα* and *IFN-γ* were up-regulated at most of time points in four immune-related organs, suggesting that the treatment of MO can induce immune responses of groupers. TLR2 and TLR5 are types of pattern recognition receptors (PRRs) for detecting various pathogen-associated molecular patterns (PAMPs) (51), and these two TLR molecules are mainly expressed on the surface of macrophages, dendritic cells, and other cells, and can activate the expression of inflammatory factors to resist pathogen invasion (24, 51, 52). Consistent with the result in the previous study (15), the expression of *TLR2* and *TLR5S* was induced by MO treatment in immune organs of fish, suggesting that the treatment of MO can promote pathogen recognition in groupers.

Serum biochemistry tests are beneficial for evaluating the health status of fish and offer a perspective on assessing the safety of pharmaceuticals. SOD and CAT play a synergistic role in the cellular antioxidant defense system (53, 54). SOD converts superoxide anions to hydrogen peroxide, and CAT rapidly breaks down the hydrogen peroxide, both of which can effectively reduce the total amount of intracellular ROS (53, 54). Lysozyme can rupture gram-negative bacteria by facilitating the breakdown of *β* (1–4)-linked disaccharides in bacterial cell wall (55). Blood serum is composed of a multitude of elements that participate in immune reactions, such as globulins and albumin (56). Our data showed that the levels of SOD, CAT, LZM, and total protein in serum were significantly up-regulated by the stimulation of MO, indicating that MO treatment can facilitate antioxidant responses and health status in fish.

In this study, the upregulation of survival rate, immune-related factors (*IL-12, TLR2, TLR5, CD4, MHC-Iα, IFN-γ*), antioxidant enzymes (CAT, SOD), lysozyme (LZM), and total serum protein (TP) were observed in groupers after injection of MO extract, which revealed that injection of these concentrations of MO may cause a moderate immunomodulatory effect rather than a pathological hyperactivation. These results may stem from the multi-target regulatory effects of MO. Bioactive constituents (e.g., magnolol) of MO may gently stimulate TLR pathways for controlling release of the pro-inflammatory factor, and simultaneously enhancing antioxidant defenses (CAT, SOD) and the antimicrobial barrier (LZM) (12, 13, 15, 18). Therefore, the treatment of MO in grouper achieves an optimal balance between enhancing disease resistance and maintaining immune equilibrium. In future research, we will optimize the frequency and dosage of MO treatment, evaluating that injection of different concentrations of MO are ultimately harmful or beneficial to grouper during *V. harveyi* infection.

In conclusion, the impact of MO as immunostimulants on the pearl gentian grouper was assessed in this study. Our data revealed that administration of MO at doses of 2, 4, 6 or 8 mg/mL can significantly improve the expression of immune-related gene expression, the CAT, LZM, SOD enzyme activity and total protein in serum, and the resistance to *V. harveyi* infection in grouper, suggesting that MO can be considered as a promising immunostimulant against *V. harveyi* infection in fish. However, further systematic investigations are warranted to comprehensively validate the biosafety and translational potential of MO in aquaculture, including but not limited to dose optimization, long-term toxicity assessment, sample size increasing, immune-related protein level verification, histopathology, intestinal flora analysis, and microbiome-host crosstalk analysis.

## Data Availability

The original contributions presented in the study are included in the article/Supplementary material, further inquiries can be directed to the corresponding author/s.
