# Supplementary material for: Magnolia officinalis enhanced immune responses and the resistance to Vibrio harveyi infection in pearl gentian groupers
Source: Front Vet Sci. 2025 Jun 18;12:1603997. doi: 10.3389/fvets.2025.1603997 (PMC12213365; doi:10.3389/fvets.2025.1603997)
Supplement: Supplementary file 1 [file Data_Sheet_1.docx]

Supplementary Table 1 Abbreviation

| Abbreviation | Meaning |
| --- | --- |
| MO | *Magnolia officinalis* |
| CAT | Catalase |
| SOD | Superoxide dismutase |
| LZM | Lysozyme |
| TP | Total protein |
| TSB | Tryptic soy broth |
| SR | Survival rate |
| qRT-PCR | Quantitative real-time PCR |

Supplementary Table 2 Survival rate between the groups

| Day (d) | PBS group | | MO2 group | | MO4 group | | MO6 group | | MO8 group | |
| --- | --- | --- | --- | --- | --- | --- | --- | --- | --- | --- |
|  | Mean (%) | SE (%) | Mean (%) | SE (%) | Mean (%) | SE (%) | Mean (%) | SE (%) | Mean (%) | SE (%) |
| 1 | 73.3 | 8.1 | 83.3 | 6.8 | 90.0 | 5.5 | 93.3 | 4.6 | 93.3 | 0.0 |
| 2 | 50.0 | 9.1 | 76.7 | 7.7 | 76.7 | 7.7 | 90.0 | 5.5 | 86.7 | 6.2 |
| 3 | 40.0 | 8.9 | 66.7 | 8.6 | 66.7 | 8.6 | 80.0 | 7.3 | 80.0 | 7.3 |
| 4 | 36.7 | 8.8 | 56.7 | 9.0 | 63.3 | 8.8 | 76.7 | 7.7 | 76.7 | 7.7 |
| 5 | 30.0 | 8.4 | 53.3 | 9.1 | 60.0 | 8.9 | 73.3 | 8.1 | 73.3 | 8.1 |
| 6 | 23.3 | 7.7 | 50.0 | 9.1 | 60.0 | 8.9 | 73.3 | 8.1 | 66.7 | 8.6 |
| 7 | 20.0 | 7.3 | 50.0 | 9.1 | 60.0 | 8.9 | 73.3 | 8.1 | 66.7 | 8.6 |
| 8 | 20.0 | 7.3 | 50.0 | 9.1 | 60.0 | 8.9 | 73.3 | 8.1 | 66.7 | 8.6 |
| 9 | 20.0 | 7.3 | 50.0 | 9.1 | 60.0 | 8.9 | 73.3 | 8.1 | 66.7 | 8.6 |
| 10 | 20.0 | 7.3 | 50.0 | 9.1 | 60.0 | 8.9 | 73.3 | 8.1 | 66.7 | 8.6 |
| 11 | 20.0 | 7.3 | 50.0 | 9.1 | 60.0 | 8.9 | 73.3 | 8.1 | 66.7 | 8.6 |
| 12 | 20.0 | 7.3 | 50.0 | 9.1 | 60.0 | 8.9 | 73.3 | 8.1 | 66.7 | 8.6 |
| 13 | 20.0 | 7.3 | 50.0 | 9.1 | 60.0 | 8.9 | 73.3 | 8.1 | 66.7 | 8.6 |
| 14 | 20.0 | 7.3 | 50.0 | 9.1 | 60.0 | 8.9 | 73.3 | 8.1 | 66.7 | 8.6 |

Values are mean ± SE.


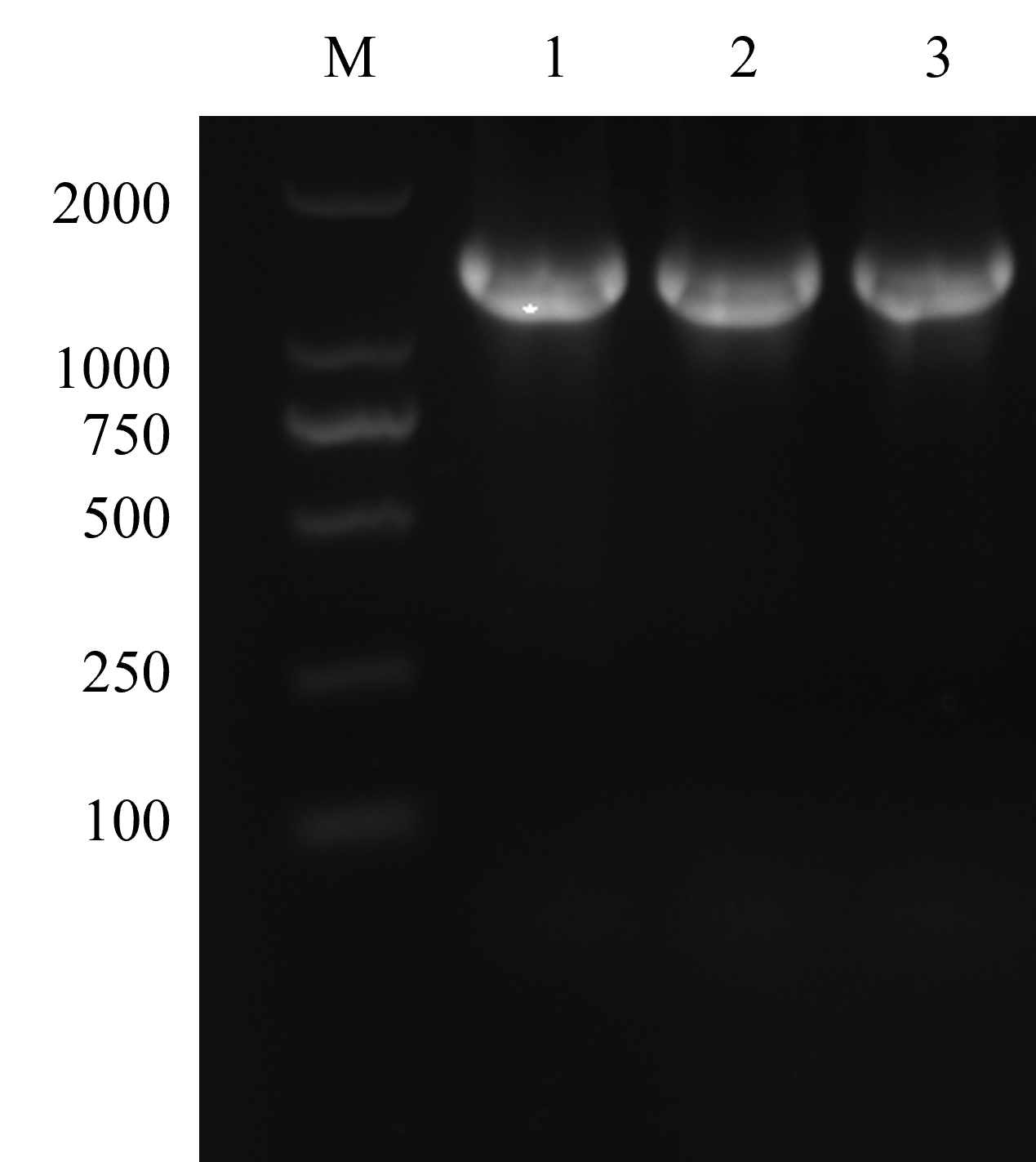


Supplementary Figure 1 *V. harveyi*-infected identification. Lane M: DL2000 DNA Marker; lane 1-3: Fragment of *V. harveyi* identified by 16s rDNA.
